# Case of Rupture of Both Ventricles of the Heart

**Published:** 1822-12

**Authors:** 

**Affiliations:** lately Physician to the Small-Pox Hospital and St. Pancras Infirmary, &c. &c.


ART. II.—*Case of Rupture of both Ventricles of the Heart.* Communicated in a Letter to Dr. MACLEOD, by Dr. ASHBURNER, lately Physician to the Small-Pox Hospital and St. Pancras Infirmary, &c. &c.

J. F. Esq. was a man of very benevolent feelings; his mind was of an unusually placid cast, and his general disposition was so gentle, that his temper was hardly ever known to be ruffled. His habit of body was robust, and his health had for a long series of years been uninterruptedly good. This gentleman was of tall stature, and large frame. A continued residence of twenty-two years in India, besides several voyages previously made to that country, had but little impaired a constitution naturally vigorous. He returned to England in 1800, and for several years enjoyed excellent health: at length he was attacked with inflammation of the eyes, accompanied by severe pain in the head. These symptoms were relieved by antiphlogistic remedies, but a permanent defect of vision remained. For several years this gentleman was occasionally subject to a flushed state of face, with some head-ach, and now and then a recurrence of inflammation in the eyes. His medical attendant had very judiciously persuaded him to insert an issue in the arm, and had enjoined particular attention to the state of his chylopoietic organs. Considerable mitigation of suffering was the natural consequence of an attention to these circumstances; but, notwithstanding all the vigilance which was employed, in the year 1818 Mr. F. suffered an attack of paralysis, that affected principally the right arm. From this complaint, however, he almost completely recovered, by attending strictly to the advice of Dr. Baillie and Mr. M. Forbes. He was cupped, and another issue was opened; a very abstemious regimen was enjoined, fish and vegetables being the principal articles of diet; a complete abstinence was observed with respect to wine and all fermented liquors. In result of this treatment, the general health became considerably improved, but the strength appeared to diminish. It was remarked by his friends, that he was more feeble, that he lost flesh, and that, in ascending a long stair-case, his respiration became affected; while a slight pallor of the countenance, with livid state of lips, accompanied a sense of uneasiness and pain in the extremities.

Still, at times, there was a determination of blood to the organs about the head, which at no time, however, affected the faculties of the mind, even in the slightest degree; but which, from local congestion, produced a flushing of the face, or an uneasy state of inflammation about the eyes. This state, when it occurred, seemed to require very little more than a degree of repose and seclusion from business, that were by no means uncongenial to the regular habits and placid disposition of this gentleman, now approaching his seventy-eighth year.

It was on Sunday, the 17th of June, 1821, that he entertained, according to his usual custom on the sabbath, a party of relations and friends at dinner; he had been in excellent spirits, and had exerted himself between the hours of divine service, which he attended twice that day, in walking a good deal; he had acquired a good appetite, and ate heartily. On Monday morning, he complained of having passed a very bad night, and he suffered from head-ach and thirst; his skin was hot and dry; the pulse was 72, full, and hard. He remarked, that he was giddy at times, and that there was "constantly a pain, not severe," (as he expressed himself,) "but troublesome, about the neck and collar-bone." This pain was worse when he endeavoured to bring down either ear towards his breast. His bowels had not been free, and he had already been directed by Mr. Houghton to take a dose of two grains of calomel, with some compound extract of colocynth. Mr. M. Forbes had wished him to lose a small quantity of blood by cupping, but he would not consent to the measure. At dinner-time he had no appetite, and took only a few spoonsful of chicken-broth, flattering himself that a light stomach, and a good night's rest, would essentially improve his condition.

On Tuesday, it was found that Mr. F. had slept better than he had done the night before, but still not well; he was hot and feverish; the sensation about the neck was more troublesome; he felt chilly; the pulse was 76, full, and hard. He had a thick yellow fur upon the tongue, which he had not the day before; he was thirsty, and had slight head-ach, with some giddiness. He consented to lose blood, and Mr. Watkins took about five ounces from the back, near the neck: some relief was obtained, but he begged that no more might be taken, as he felt faint. The frequency of the pulse after the cupping was exactly the same as before, but the hardness of the beat was considerably lessened. Two hours afterwards, he remarked that he had never been so little weakened by the loss of blood. The medicine having had no effect, some castor-oil was prescribed. In the evening, the pulse was at 78; it had a jarry beat. Some alvine evacuations procured a good night; but on Wednesday morning the pulse continued the same, as to number and character. The feelings of the patient were, however, improved: he said he felt much better; his head was lighter; and he should have thought himself well in every respect, if he had not still the uncomfortable sensation at the sides of his neck, whenever he moved his head. In the course of the morning, the pulse got up to 82. It was observed by some of his friends that his sensibility to external impressions became more acute than usual, and that his power of vision seemed to be improved. A little past six in the evening, after dinner, at which he ate but little, the pulse was 92.

About nine o'clock, having just left the water-closet, he was heard to call his servant, in a voice more than usually loud and urgent. The man immediately hastened to him, and found him sinking into a fainting-fit: he was carried into the dining-room; the head hung forward; the face was livid; and the pulse was almost imperceptible. He was breathing with stertor; the extremities were becoming cold. The head being supported, the shirt-collar loosened, the hands being rubbed, and the feet immersed in a tepid bath, a few teaspoonsful of warm port wine and water were administered, and he became better, signifying a wish to be taken to his bed-room. In the attempt to remove him, the faintness recurred, the head fell forward, and the breathing was again stertorous; the pulse was quite gone. A teaspoonful of warm wine and water had the effect of restoring him: he was sensible, and endeavoured to talk, but his speech was almost inarticulate. The servants had prepared a bed in the room, and he was lifted into it: here, for a short time, he appeared better, and was engaged in fervent prayer. Upon being supported to take some fluid, he did not readily swallow it; he coughed, and endeavoured to inspire, gasping repeatedly with a loud and distressing sound; but the effort was too much for him, and, falling back, he expired, about an hour from the period of the first fainting-fit.

Forty-three hours after death the body was examined, in presence of Dr. James Forbes and myself, by the late Mr. Gatcombe. Its external aspect conveyed an idea of the great muscular power this gentleman must have possessed in his youth. There was a remarkably broad and deep chest, constituting part of a trunk, the muscles of which, in connexion with the neck and limbs, were well set; the arms and legs, though now thin, were well proportioned, and had evidently been stout and muscular.

The head was first examined. It was found that the bony structure of the skull was unusually thick; the furrows of the meningeal arteries on its internal surface were deep and large.

The dura mater adhered firmly to the bone in every part. The arachnoid membrane and the pia mater indicated a state of chronic inflammation: they were considerably thickened, and separated from each other by a considerable effusion of yellow serous fluid. The substance of the brain was every where soft and pulpy; its blood-vessels were empty; the arteries, throughout the cavity of the cranium, were considerably ossified. There was a small cyst, apparently of long standing, about the size of a garden-pea, in the pons varolii.

In the chest, the lungs were healthy, but they were loaded with venous blood. The pericardium was found to be full of blood, to the amount of about twelve ounces; a small part only of this blood was coagulated. Upon the anterior surface of the left ventricle of the heart, there were two or three small openings, which admitted the passage of a probe into its cavity, where there was a quantity of uncoagulated blood. On the posterior part of the right ventricle, near its junction with the left, was a jagged opening, sufficiently large to admit the passage of the fore-finger into its cavity, which was quite empty. The muscular structure of the heart was much thinner than it is in the healthy organ; the walls of the right ventricle, indeed, being not a third part of their usual thickness: they were very friable, and tore like wetted brown paper.

The aorta was enlarged, from its junction with the heart, along the course of the arch; and behind its inner coat were numerous spots of ossification.

The abdomen was not examined; and this is the more to be regretted, as it would have been a matter of some interest to have ascertained the state of the liver in a person who had passed so many years in the East Indies.

The pathological considerations which suggest themselves upon this case, relate principally to the altered condition of the muscular system in an individual who had attained his seventy-eighth year. There is no doubt that the determination to disease commenced in the head; and this was indicated by the repeated inflammations which took place in the eyes, the flushed state of face, and accompanying head-aches; but, above all, by the evidence of the long-continued chronic action, afforded by the thickened membranes of the brain and parietes of the cranium, and by the paralysis which took place a few years before death.

In an aged individual, in whom the tendency to apoplexy had been combatted by the most vigilant attention to diet and regimen, it was natural to expect that the absorptive action, forming a part of the process known to physiologists by the term decay, should affect the whole muscular system of the body; and the substance of the heart participating in its consequences, would gradually become thinner and more friable, so that a time would arrive when its strength would be inadequate to its functions. In the present case, the conjecture is that the muscular substance of the left ventricle first gave way; the apertures from its cavity were very small, and, though the escape of blood through them caused repeated faintings, yet death did not take place till an obstruction occurred in the passage of the blood through the lungs. The left ventricle, after repeated ruptures, was becoming incompetent to the aortic circulation; and, in the mean time, the venous blood sent from the right side of the heart accumulated in the lungs; the right ventricle, acting to propel a further quantity against this accumulation, at length gave way, forming a large rent. Corvisart* notices the fact, that the rupture of the heart is not always attended with sudden death, because, he remarks, that the blood may escape in some cases through a very narrow and oblique opening, and consequently slowly, and in small quantities. He had never himself witnessed a case of ruptured heart, and notices none where both ventricles gave way.

Morgagni relates instances of persons dying from ruptures of one ventricle; in some of them the right, and in others the left, had burst: but he has no example of the two having been lacerated in one individual; nor am I aware of any such having been placed upon record.

A very remarkable circumstance in the case of Mr. F. is the gradual increase in the number of the arterial pulsations, evincing the existence of a strong determination in the system for some days, which it would be difficult to explain, but which, if repeatedly observed, might lead to inferences highly useful to diagnosis.

* Troisieme edition, 1818.

